# Microtomographic Evaluation of Canal Centralization and Dentine Removal after Canal Preparation with Two Rotary Systems: HyFlex EDM and ProTaper Next

**DOI:** 10.1055/s-0041-1739440

**Published:** 2022-01-11

**Authors:** Vivian Ronquete, Alexandre Sigrist de Martin, Karin Zuim, Thais Machado de Carvalho Coutinho, Eduardo Fagury Videira Marceliano, Paula Avelar Da Silva Ribeiro Goulart, Ricardo Tadeu Lopes, Carlos Eduardo Da Silveira Bueno, Marília Fagury Videira Marceliano-Alves

**Affiliations:** 1 Department of Endodontics and Dental Research, Iguaçu University, Nova Iguaçu, Brazil; 2 Department of Endodontics, São Leopoldo Mandic University, Campinas, Brazil; 3 Dental Clinic Department, Brazilian Army General Hospital of Belem, Belem, Brazil; 4 Laboratory for Nuclear Instrumentation, Federal University of Rio de Janeiro, Rio de Janeiro, Brazil

**Keywords:** micro–computed tomography, nickel–titanium alloy, apical transportation, ProTaper Next, HyFlex EDM

## Abstract

**Objective** This study compared the ProTaper Next (PTN; Dentsply Sirona, Tulsa, Oklahoma, United States) and HyFlex EDM (HEDM; Coltene/Whaledent AG, Alstätten, Switzerland) systems using micro–computed tomography (CT).

**Materials and Methods** Twenty-one mesial roots classified as Vertucci's type IV from extracted mandibular first molars with curvatures between 20 and 40 degrees were selected. The teeth were scanned using a micro-CT before and after root canal preparation by both systems, applied to the same root, in alternating canals. The following parameters were analyzed: canal centering, apical transportation, root canal diameter/root diameter.

**Results** No statistically significant differences between both systems were observed for any of the assessed morphological parameters ( *p* > 0.05). All canals presented diameter enlargement of more than 40% in relation to root diameter in the cervical and middle segments. No statistically significant difference was noted between the HEDM and PTN groups. The wear percentage for the HEDM group in the cervical and middle thirds were 49.66 ± 8.65 and 46.48 ± 14.29, respectively, and 51.02 ± 11.81 and 45.48 ± 10.79 for the PTN group, respectively.

**Conclusion** Both systems displayed similar mandibular molar mesial canals preparation, with no differences noted for any of the assessed parameters. Both groups showed increased canal diameter in the cervical and middle thirds by more than 40%.

## Introduction

Understanding the anatomy of tooth canals is an essential step for effective endodontic therapy. Studies report changes to root canal morphology during canal preparation, which may vary according to applied instrumentation technique. 1 In addition, root curvature introduces a complexity that influences the ability of instruments to prepare and clean all root canal walls, regardless of the applied instrumentation system. 2 3 These difficulties in preparation may predispose the root canal to persistent infection, as bacteria may remain in unprepared areas, adhered to canal walls or in areas inaccessible to the chemical–mechanical preparation, which can lead to failures in endodontic treatment. 4

Automated instruments made from a nickel–titanium (NiTi) alloy have become widely applied in clinical practice. The NiTi alloy presents a lower elasticity modulus than stainless steel, allowing for easy and efficient preparation of curved root canals. 5 6 However, errors in iatrogenic procedures, such as deviations, perforations, or root canal transportation, may occur due to the applied instrumentation technique, particularly in curved canals, culminating in deviations of the original root canal pathway. 7 8

As a way of controlling these factors, HyFlex EDM (HEDM; Coltene/Whaledent AG, Alstätten, Switzerland) instruments have been proposed, manufactured with a controlled memory alloy using the electric discharge machining technology. This manufacturing process improves the fracture strength and efficiency of the cutting blade. 9 10 11 Another system comprises the Protaper Next (PTN; Dentsply Maillefer, Ballaigues, Switzerland), manufactured with lower mass in its M-Wire alloy (containing martensite portions in its microstructure) which, through a heat treatment process, provides greater flexibility while maintaining cutting efficiency and higher resistance to cyclic fatigue when compared with the conventional NiTi alloy. 3 12

The vast majority of the preparation evaluation methods described earlier only assess two-dimensional changes. However, root canal anatomy is altered in three dimensions (3D) during chemical–mechanical preparation. 1 Studies have applied micro–computed tomography (CT) to evaluate the chemical–mechanical preparation ability of different endodontic instruments. This type of methodology displays advantages over other methodologies, mainly because it is nondestructive. 13 14 In addition, the risk of root fracture is greater when the canal diameter is widened by more than 40% of the root width. 15

In this context, the aim of the present study was to compare the modeling ability of the PTN (Dentsply Sirona, Tulsa, Oklahoma, United States) and HEDM (Coltene/Whaledent AG) instrument systems in mandibular molar mesial canals using micro-CT as an evaluation method.

## Materials and Methods

### Sample Preparation and Selection

This study was approved by the local ethics committee under CAAE number 79232617.7.0000.5374. Twenty-one teeth (based on a sample calculus) were selected from an initial sample of 122 mandibular human molar teeth, displaying complete rhizogenesis, patent canals presenting mesial roots, individualized canals and foramina, Vertucci's type IV classification, and root curvature between 20 and 40 degrees. 16 Tooth crowns were removed by a diamond disc to standardize canal length at 16 mm. The 16 mm was divided into three thirds. The apical third considered at 1 to 5 mm, the middle at 6 to 10 mm, and the cervical at 11 to 16 mm from the apex. After specimen selection and standardization, the specimens were submitted to an initial scanning using a SkyScan 1173 device (Bruker-microCT, Kontich, Belgium) at 17.09 μm pixel size, 114 mA, 70 kV, 360 degrees, 1.0 for ∼18 minutes per specimen.

The acquired images were reconstructed in transverse slices using the NRecon 1.7.1.0 software (Bruker-microCT). The 3D images of the mesial roots were obtained and evaluated by the CTVol v.2.2.1 software (Bruker-microCT). The internal morphology of the Vertucci's type IV root canal was confirmed by the micro-CT images. Canal morphological parameters (volume and surface area) were acquired using the CTAn v.1.14.4 software (Bruker-microCT) and served as basis for sample matching.

### Canal Preparation

A single experienced operator performed the setup for both systems. The instrumentation was applied to a same root, in alternating mesial canals. The working length was determined as 1 mm below the foraminal constriction. The instruments used in the PTN system were as follows: SX-cervical, X1 - 17.04 middle and apical thirds, and X2 - 26.06 in the apical third, driven by the VDW Gold engine at 300 rpm and 2 NCm, according to the manufacturer's instructions. The HEDM system followed the sequence: 25.12, cervical third, 10.05 and 25.08 files, in the middle and apical thirds, at 500 rpm and 2.5 NCm (25.12 and 25.08) and 300 rpm and 1.8NCm (10.05) respecting the manufacturer's instructions. Each file made three in-and-out movements in each canal and each instrument was used once per canal and then discarded, in both groups. At each instrument change, each canal was irrigated with 10 mL of a 2.5% NaOCl solution using a 30G needle. For smear layer removal, 3 mL of a 17% EDTA solution was used for 1 minute and a final irrigation was applied using 2.5 mL of a 2.5% NaOCl solution, totaling 25 mL of NaOCl 2.5% per root. 17 18 19

### Analysis of Postinstrumentation Images—Micro-CT Evaluation

After preparation, the roots were subjected to a new micro-CT scan using the same parameters described previously. The following morphometric parameters were analyzed: canal centralization after chemical–mechanical preparation, apical transportation, and canal and root widths. The 3D Slicer 4.4.0 software (available at http://www.slicer.org ) was used to coregister the 3D models of the pre- and postoperative phases.

### Canal Transportation and Centralization

**Fig 1:**
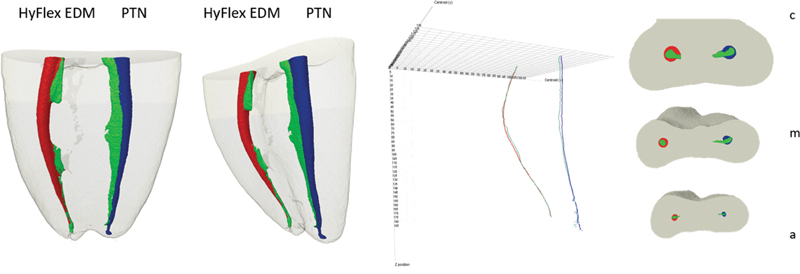
(A) Three-dimensional images and diagram displaying the combination of the central axis of the HyFlex EDM (HEDM) preinstrumentation (green line) and postinstrumentation (red line) root canals and ProTaper Next (PTN) preinstrumentation (green line) and postinstrumentation (blue line) root canals. (B) Cross-sections representative of overlapping root canals before (green) and after (blue) HEDM and PTN preparation, in the cervical (c), middle (m), and apical (a) thirds.

Using the centralization data extracted from the CTAn program (Bruker-microCT), the XLSTAT-3DPLOT for Windows plugin for Excel (Addinsoft, New York, United States) was used to elaborate a center of gravity variation diagram connected along the *Z* -axis. Root canal transportation was evaluated from the center of gravity variation (in mm), comparing the centers of gravity before and after preparation for all radicular, cervical, middle, and apical canal segments. Representative measurements were also presented graphically in the form of diagrams ( Fig. 1 ).

### Canal/Root Width and Potential Risk for Root Fracture

**Fig 2:**
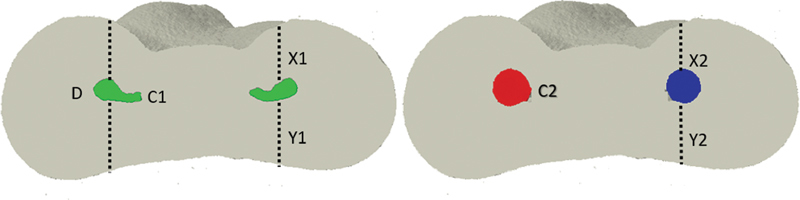
Representative mandibular molar root shape showing the unprepared (left) canal diameter, with X1 and X2 representing dentin thicknesses in the inner wall of the root and Y1 and Y2 representing dentin thicknesses in the outer wall. D represents the dentin diameter, and C1 and C2 represent the canal diameters before and after preparation.

The CTAn software v.1.14.4 (Bruker-microCT) was used to measure the diameter of the pre- and postoperative canals in relation to root diameter at the cervical, middle, and apical levels, as well as the variations in canal diameter in relation to the width of the external dentin, as per the method reported by Gambill et al, with adaptations, 20 in which the apical third was considered at 1 to 5 mm, the middle at 6 to 10 mm, and the cervical at 11 to 16 mm from the apex. Extensions were determined by measuring the shortest distance from the edge of the unprepared canal to the tooth border, both in the mesial and distal directions, and then comparing with the same measurements obtained from the treated canal images. The following formula was used: ([X1 − X2] − [Y1 − Y2]) × X1. The distance represented by X1 was measured before instrumentation, from the edge of the canal to the edge of the root in the mesial region, and Y1 represents the distance from the canal to the root border in the distal region before instrumentation. The distance represented by Y1 was measured from the edge of the canal to the edge of the root in the middle region, after instrumentation. Y2 represents the distance from the canal to the root border in the distal region after instrumentation. The canal diameter variation in relation to external dentin was measured by tracing a straight line from the middle border to the distal border, followed by initial canal diameter and diameter after canal enlargement assessments. A potential fracture risk was considered if the diameter of the postoperative canal corresponded to more than 40% of the root width 15 20 21 ( Fig. 2 ).

### Statistical Analyses

Data distribution was analyzed by the Shapiro–Wilk's normality test. Intragroup and intergroup analyses were performed by applying the unpaired *t* -test. The analysis of variance test was used for intragroup comparisons. The level of significance was set at 5% for all statistical tests ( *p* < 0.05).

## Results

### Canal Transportation and Centralization

Variations in the center of gravity were not statistically different between the groups. A statistically significant difference in the HEDM group for the middle and apical levels was observed. The results of root canal transportation and centralization are summarized in Table 1 and Fig. 1 .

**Table 1:** Transport and centralization of the mandibular molar mesial canals after preparation by the assessed systems

HyFlex EDM				ProTaper Next		
Level	Mean ± SD	Median	Range	Mean ± SD	Median	Range
Cervical	0.65 ± 0.38 ^**aA**^	0.72	0.05–1.15	0.54 ± 0.37 ^**aA**^	0.51	0.02–1.07
Middle	0.47 ± 0.26 ^**abA**^	0.47	0.05–0.98	0.47 ± 0.34 ^**aA**^	0.48	0.02–0.98
Apical	0.32 ± 0.18 ^**bA**^	0.32	0.03–0.69	0.32 ± 0.16 ^**aA**^	0.34	0.08–0.6
Total	0.40 ± 0.22 ^**A**^	0.36	0.04–0.88	0.52 ± 0.22 ^**A**^	0.58	0.01–0.79

Abbreviation: SD, standard deviation.

Note: Different lowercase letters represent significantly intragroup differences. Different uppercase letters represent significant differences between the groups.

### Canal/Root Width and Potential Risk of Root Fracture

**Fig 3:**
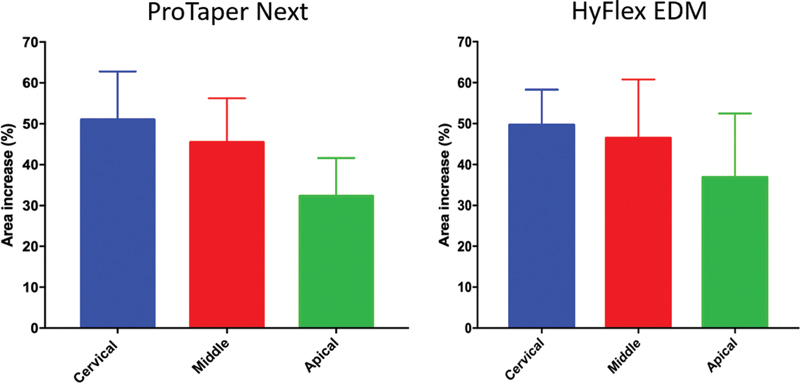
Canal/root width and potential risk of root fracture after preparation by the assessed systems.

The width of the root canal in relation to root width was significantly increased by both systems ( *p* < 0.05) ( Table 2 ). No statistically significant differences between the groups was observed regarding the root canal and root width relationship, comparing cervical, middle, and apical levels before and after preparation ( *p* > 0.05). The cervical and middle thirds of the canal diameter were increased by more than 40% of the root width in both groups. Root diameter enlargement was not more than 40% in the apical third ( Figs. 2 and 3 ; Table 2 ).

**Table 2:** Canal/root diameter ratio and potential risk of root fracture after preparation by the assessed systems

Section	HyFlex EDM			ProTaper Next		
	Mean ± SD	Median	Range	Mean ± SD	Median	Range
Cervical canal (mm)	1.40 ± 0.94 ^**aA**^	1.13	0.32–3.83	0.94 ± 0.69 ^**aA**^	0.85	0.22–3.34
Middle canal (mm)	1.25 ± 0.91 ^**aA**^	1.00	0.14–4.00	0.97 ± 0.76 ^**aA**^	0.92	0.00–2.84
Apical canal (mm)	1.16 ± 0.81 ^**aA**^	1.12	0.08–2.86	0.76 ± 0.48 ^**aA**^	0.58	0.00–1.80
Cervical dentin (%)	49.66 ± 8.65 ^**aA**^	50.00	32.37–65.03	51.02 ± 11.81 ^**aA**^	47.34	30.14–74.86
Middle dentin (%)	46.48 ± 14.29 ^aA^	43.28	24.69–80.89	45.48 ± 10.79 ^**aA**^	40.16	29.84–74.40
Apical dentin (%)	36.85 ± 15.64 ^**bA**^	33.26	17.86–86.30	32.29 ± 9.33 ^**bA**^	27.24	15.29–51.8

Abbreviation: SD, standard deviation.

Note: Different lowercase letters represent significantly intragroup differences. Different uppercase letters represent significant differences between the groups.

## Discussion

Root canal preparation includes both root canal system enlargement and modeling, along with disinfection. A variety of instruments and techniques have been developed and described for this critical root canal treatment phase. Although many root canal preparation reports are found in the literature, definitive scientific evidence on the quality and clinical suitability of different instruments and techniques remains undefined. 22 Micro-CT images have been used to assess the chemical–mechanical preparation capacity of different endodontic instruments. This methodology presents advantages over other methodologies, mainly because it is nondestructive. 13 14

The present study compared the centralization capacity of preparations using two rotary systems. The HEDM system is a memory-based file system, manufactured by electrical discharge machining that allows for high fatigue strength and greater flexibility. It comprises varied triangular conicity in its cervical portion, is trapezoidal in its middle portion, and quadrangular in the apical portion. The PTN file system presents variable conicity (increasing and decreasing in the apical to cervical direction, with fixed X1 and X2, an eccentric rectangular cross-section and is composed of the M-Wire alloy).

Canal transportation was evaluated based on center of gravity variations and, although the HEDM and PTN systems do not share similarities in alloy metallurgy, file and tip geometry, and diameter in D0, the results obtained herein indicated no significant differences in relation to the morphological parameters assessed after canal preparation. Both groups maintained preparation centralization. The similar results observed for both systems may be due to the alloy metallurgy of each system, as both undergo alloy heat treatment, allowing for greater flexibility and preparation centralization. 12 13 These results are in agreement with other studies assessing canal centralization. 12 23

Root canal diameter and root in relation to dentin wear in mandibular molar mesial canals were evaluated. Dentin thickness evaluations are essential, considering that excess dentin removal can predispose tooth root fractures. 15 21 24 25 The HEDM and PTN systems increased the cervical and middle thirds of the canals by more than 40%. This may have occurred because both files used in the cervical and middle thirds, HEDM 25.12 and SX (19/.035), share the same cervical portion geometry, a triangular cross-section. 26

Although no statistically significant difference was observed between the groups, the PTN system led to an increased cervical third in comparison to the HEDM system. This difference may have been caused because the file used in the PTN group to prepare the cervical third was the SX (19/.035) file, prepared with a conventional NiTi alloy, as part of the ProTaper Universal file system. Conventional NiTi alloy as compared with heat-treated or memory-controlled alloys tends to further increase the canal diameter and further decentralize the preparation. 6 23 24 25 26 27 This study indicates contrasting results when compared with other studies that both shared 21 and did not share 15 20 28 29 30 the same methodology.

## Conclusion

No differences in the assessed morphological modeling parameters were observed for both root preparation systems. Both groups maintained the preparation centralization. The assessed systems increased the cervical and middle thirds of the root canals by more than 40%, increasing potential fracture risks due to excessive dentin removal.
